# Flame-retardant properties and mechanism of LGF/PBT/DOPO-HQ-conjugated flame-retardant composites

**DOI:** 10.3389/fchem.2022.981579

**Published:** 2022-10-12

**Authors:** Junzhuo Sun, Daohai Zhang, Xiaoyu Shang, Fang Tan, Dongmei Bao, Shuhao Qin

**Affiliations:** ^1^ School of Chemical Engineering of Guizhou Minzu University, Guiyang, China; ^2^ Polymer Composites Engineering Research Center of Guizhou Minzu University, Guiyang, China; ^3^ National Engineering Research Center for Compounding and Modification of Polymer Materials, Guiyang, China

**Keywords:** conjugated flame retardant, polybutylene terephthalate, flame-retardant mechanism, composites, gas-phase flame retardant

## Abstract

In this article, long fiber reinforced polybutylene terephthalate (LGF/PBT/DOPO-HQ) flame-retardant composites were prepared using 10-(2,5-dihydroxy phenyl)-10H-9-oxa-10-phosphaphenanthrene-10-oxide (DOPO-HQ) as the conjugated flame-retardant. The effects of different flame-retardant contents on the combustion properties of the composites were investigated. The results showed that after adding 14% of DOPO-HQ, the flame-retardant effect of the composite reached the V-0 level of UL-94 fire rating with an ultimate oxygen index (LOI) of 26.4%. The average heat release rate (Av-HRR), peak heat release rate (PHRR), and total heat release rate (THR) decreased by 45.9, 56.5, and 32.6%, respectively. This shows that LGF/PBT/DOPO-HQ composite has good flame-retardant properties. Meanwhile, the flame-retardant mechanism of cohesive phase and gas-phase synergy during the combustion of flame retardants was analyzed by carbon layer morphology and dynamic thermal decomposition.

## Introduction

Polybutylene terephthalate (PBT) is an engineering plastic with excellent all-around properties that is used in many areas, such as home electronics, automotive industry, and transportation construction. In practice, it is relatively common to enhance the performance of PBT materials with more cost-effective glass fibers to obtain higher performance. However, PBT resins are extremely flammable with a limiting oxygen index (LOI) of 20–22%, generating large amounts of heat during combustion accompanied by severe melt dripping. This makes them extremely dangerous for fire safety in applications (Zhu et al., 2017; Niu et al., 2018). Among the glass fiber reinforced PBT composites, long glass fiber reinforced PBT composites can achieve higher strength, modulus, dimensional stability, and thermal stability than short glass fiber reinforced PBT composites (Cai et al., 2015; Suzanne et al., 2017). However, LGF/PBT composites are still very flammable, so they need to be modified with flame retardants.

Phosphorus is an effective flame-retardant element, and phosphorus-based flame retardants can thermally decompose and release phosphorus-containing reactive radicals (e.g., PO2-, PO-, and HPO-, etc.) in the gas phase when used (Zhang et al., 2022a; Zhang et al., 2022b). The average reactivity of these radicals is 10 times that of Cl- and 5 times that of Br-, which can effectively trap hydroxyl and hydrogen radicals, terminate the radical segment, and prevent further thermal decomposition of polyester materials. Among the phosphorus-based flame retardants, numerous derivatives of 9,10-dihydro-9-oxo-10-phosphofi-10-oxide (DOPO) have attracted widespread attention for use as flame retardants (Brehme et al., 2011; Liu et al., 2013; Battig et al., 2021). Therefore, in this study, 10-(2,5-dihydroxyphenyl)-10H-9-oxo-10-phosphophenanthrene-10-oxide (DOPO-HQ) was used as a flame retardant for PBT.

## Materials and methods

### Materials

The PBT that was as used in this study was obtained from the Chinese Petrochemical Corporation. Glass fiber without alkali was bought from JU SHI Limit Co., China. Meanwhile, the DOPO-HQ was obtained by Chinese Wan Sheng Technology Co., Ltd.

### Preparation of LGF/PBT/DOPO-HQ retardant composites

Polybutylene terephthalate (PBT), glass fibers (GFs), and 10-(2,5-dihydroxy phenyl)-10H-9-oxo-10-phosphofi-10-oxide (DOPO-HQ) were mixed in a twin-screw extruder (Model TSE-40A/400-44-22, Kobelco Machinery, Nanjing, China). The temperatures in six different zones from the hopper to the die were 210, 215, 220, 225, 230, and 235°C. The screw’s speed was 200 rpm and the impregnation temperature was 26°C. The obtained composites were dried in a vacuum oven at 90°C for 8 h. They were then molded (CJ80M3V type, Zhende Plastic Machinery, Guangdong, China) into various specimens at 245°C for testing and characterization. Each group of composites was added with 20 wt% LGF. Six samples with DOPD-HQ weight ratios of 0 wt%, 7.5 wt%, 10 wt%, 12.5 wt%, 14 wt% and 15 wt% were coded as LGF/PBT, LGF/PBT/DOPO-HQ-7.5, LGF/PBT/DOPO- HQ-10, LGF/PBT/DOPO-HQ-12.5, LGF/PBT/DOPO-HQ-14, and LGF/PBT/DOPO-HQ-15, respectively.

### Characterization

To characterize the prepared samples, UL-94, vertical combustion test, cone measure heat, LOI, scanning electron microscopy (SEM), thermogravimetric-Fourier transform infrared spectroscopy (TG-FTIR), and tensile and flexural tests were performed.

## Results and discussion

### Flame-retardant properties of LGF/PBT/DOPO-HQ-conjugated flame-retardant composites

**FIGURE 1:**
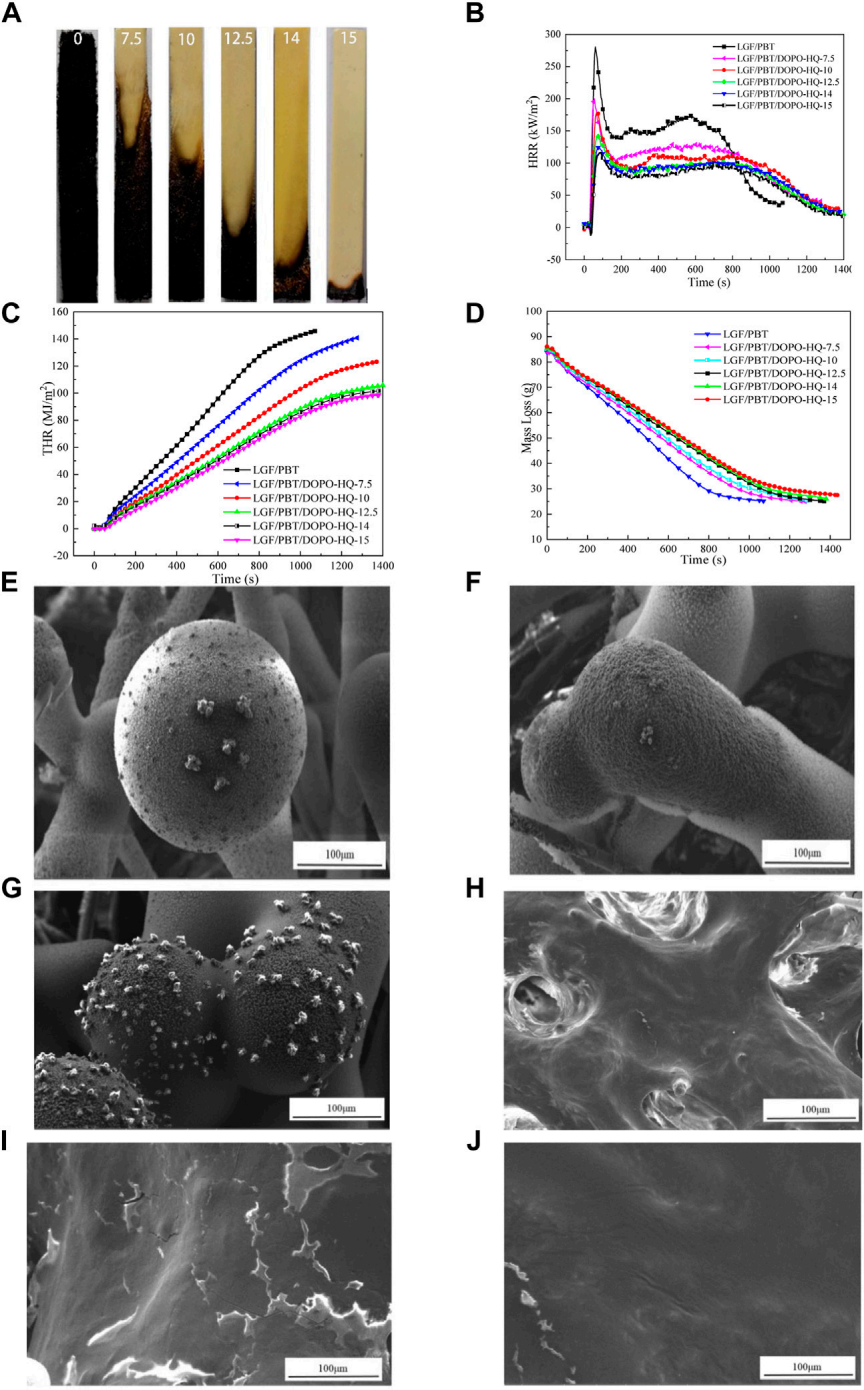
**(A)** The pictures of LGF/PBT/DOPO-HQ-conjugated flame-retardant composites after UL-94 testing: (DOPO-HQ: 0;7.5;10;12.5;14; 15%); **(B**–**D)** the curves of LGF/PBT/DOPO-HQ-conjugated flame-retardant composites at an external heat flux of 50 kW/m2 as a function of burning time: **(B)** HRR; **(C)** THR; **(D)** Mass Loss; **(E**–**J)** the microstructure (1000 magnification) of the burning surface of LGF/PBT/DOPO-HQ-conjugated flame-retardant composites. **(E)** LGF/PBT; **(F)** LGF/PBT/DOPO-HQ-7.5; **(G)** LGF/PBT/DOPO-HQ-10; **(H)** LGF/PBT/DOPO-HQ-12.5; **(I)** LGF/PBT/DOPO-HQ-14; **(J)** LGF/PBT/DOPO-HQ-15.

The flammability (UL-94, LOI) results of all of the samples are summarized in Table 1. From this table, it can be seen that the addition of LGF prevents the PBT matrix resin from producing melt drops during combustion. When the content of DOPO-HQ is increased, the vertical burning time of the LGF/PBT/DOPO-HQ-conjugated flame-retardant composite gradually decreased, and the LOI value of the flame-retardant composite (DOPO-HQ-7.5) increased from 22.7 to 27.1%. In addition, when the content of DOPO-HQ reached 14 wt%, the LGF/PBT/DOPO-HQ-15-conjugated flame-retardant composite achieved UL-94 V-0 rating and the LOI value increased from 21.2 to 26.4%. Figure 1A shows the macroscopic morphology of LGF/PBT/DOPO-HQ-conjugated flame-retardant composites after UL-94 testing. According to Figure 1, the burning length of LGF/PBT/DOPO-HQ-conjugated flame-retardant composites decreases as the DOPO-HQ content increases. These results prove that DOPO-HQ has an efficient flame-retardant effect on LGF/PBT composites.

**TABLE 1:** The flame-retardant properties of LGF/PBT/DOPO-HQ-conjugated flame-retardant composites.

Samples	UL-94			LOI (%)
	t1/t2	Dripping	Ranking	
LGF/PBT	BC	No	HB	21.2
LGF/PBT/DOPO-HQ-7.5	BC	No	HB	22.7
LGF/PBT/DOPO-HQ -10	8.2/22.6	No	V-1	24.3
LGF/PBT/DOPO-HQ -12.5	7.9/14.7	No	V-1	25.8
LGF/PBT/DOPO-HQ -14	4.2/5.3	No	V-0	26.4
LGF/PBT/DOPO-HQ -15	3.5/4.6	No	V-0	27.1

Note: t1 and t2 are the burning time in the UL-94, test (unit: second).

### The combustion properties of LGF/PBT/DOPO-HQ-conjugated flame-retardant composites

The combustion behavior of the materials is shown in Figures 1B,C, which illustrate the relationship between HRR and THR with respect to the burning time, respectively. PHRR is an important parameter for assessing the fire risk. According to Figure 1B, after ignition, a peak PHRR immediately appears on the HRR curve of the LGF/PBT/DOPO-HQ-conjugated flame-retardant composites. After the peak PHRR, a “plateau zone” appears and then gradually decreases. With the increase of DOPO-HQ addition, the HRR curve of the LGF/PBT/DOPO-HQ-conjugated flame-retardant composites and PHRR gradually decreased and the width of the platform zone gradually widened. This indicates that the addition of DOPO-HQ helps LGF/PBT composites to form a special carbon layer structure. This can play a role in heat insulation and oxygen barrier, and help to improve the flame-retardant effect of the system (Gallo et al., 2011; Liu et al., 2011). This behavior suggests that DOPO-HQ plays an important role in the flame retardancy of LGF/PBT composites, which is a condensed phase flame-retardant effect.

According to Figure 1C and Table 2, with the increase of DOPO-HQ addition, the PHRR, HRR, THR, maximum average heat release rate (MAHRE), and fire growth rate (FIGRA) of the flame-retardant composite system decreased. When the content of DOPO-HQ was 14%, the PHRR, HRR, THR, MAHRE of the conjugated flame-retardant composite of LGF/PBT/DOPO-HQ-14, and FIGRA values decreased by 56.5% (125.4 kW/m^2^), 45.9% (75.3 kW/m^2^), 32.6% (98.1 kW/m^2^), 48.3% (83.4 kW/m^2^), and 67.5% (1.56), respectively. When DOPO-HQ was added at 15%, LGF/PBT/DOPO-HQ-15 flame retardants PHRR, HRR, THR, MAHRE, and FIGRA reduced 59.3% (117.4 kW/m^2^), 47.7% (72.9 kW/m^2^), 33.7% (96.5 kW/m^2^), 49.5% (81.4 kW/m^2^), and 69.4% (1.47), respectively. This indicates that DOPO-HQ has a high flame-retardant property for LGF/PBT composites. Meanwhile, the flame-retardant effect of LGF/PBT/DOPO-HQ-conjugated flame-retardant composites is better when the DOPO-HQ content is higher.

**TABLE 2:** The combustion results of LGF/PBT/DOPO-HQ composites obtained from CONE.

Samples	TTI (s)	Av-HRR (kW/m^2^)	PHRR (kW/m^2^)	THR (MJ/m^2^)	TSR (m^2^/m^2^)	Av- EHC (MJ/m^2^)	FIGRA (kW/m^2^ • s)	MAHRE (kW/m^2^)	CO yield (kg/kg)
LGF/PBT	23	139.3	288.5	145.6	2904	22.07	4.80	161.2	0.056
LGF/PBT/DOPO-HQ-7.5	18	116.0	209.2	140.6	2911	21.27	3.80	127.8	0.075
LGF/PBT/DOPO-HQ-10	20	91.5	177.4	123.1	3442	18.14	2.73	103.7	0.089
LGF/PBT/DOPO-HQ-12.5	21	79.3	137.5	104.5	3585	16.73	1.96	92.8	0.091
LGF/PBT/DOPO-HQ-14	22	75.3	125.4	98.1	3667	15.41	1.56	83.4	0.104
LGF/PBT/DOPO-HQ-15	22	72.9	117.4	96.5	3784	15.20	1.47	81.4	0.109

TTI: time to ignition.

It can be seen in Table 2 that there is a huge increase in total smoke release (TSR) and CO production, and a moderate decrease in average effective heat of combustion (Av-EHC) for the conjugated flame-retardant composites compared to PBT. In this case, Av-EHC is used to evaluate the heat generated by the volatiles from polymer cracking during combustion. This value is greater if the combustion is more complete. Both TSR and CO yields of the LGF/PBT/DOPO-HQ-conjugated flame-retardant composites increased as the amount of DOPO-HQ added increased and the Av-EHC of the LGF/PBT/DOPO-HQ-conjugated flame-retardant composites decreased. This indicates that the effective combustion component of the conjugated flame-retardant composites in the gas phase decreases during combustion (Van Der Veen and De Boer, 2012). This also suggests that when burning LGF/PBT/DOPO-HQ-conjugated flame-retardant composites, DOPO-HQ may release phosphorus oxygen radicals (PO-) in the gas phase, trap hydrogen radicals (-H) and hydroxyl radicals (-OH), terminate chain radicals, play a role in gas-phase flame retardation, and eventually lead to incomplete combustion of the effective burning component in the gas phase (Laoutid et al., 2009). This is consistent with the literature, which reports that DOPO-HQ plays a major role in gas-phase flame retardancy in polyester processes. Furthermore, according to Figure 1D, the residual amount of conjugated flame-retardant composites increased with the increase of DOPO-HQ addition. This proves that the addition of DOPO-HQ contributes to the formation of LGF/PBT/DOPO-HQ-conjugated flame-retardant composites during combustion. The carbon layer has a certain condensed phase flame-retardant effect on the LGF/PBT/DOPO-HQ-conjugated flame-retardant composite, and so it achieves an excellent flame-retardant effect.

### Carbon layer morphology of LGF/PBT/DOPO-HQ-conjugated flame-retardant composites

Based on the aforementioned cone-combustion performance analysis, we found that DOPO-HQ has condensed phase flame-retardant properties for LGF/PBT/DOPO-HQ flame-retardant composites. Therefore, the microstructure of carbon layer of LGF/PBT/DOPO-HQ-conjugated flame-retardant composites after combustion was observed. From Figures 1E–J, it can be seen that with the increase of DOPO-HQ addition, the carbon formation performance on the surface of LGF/PBT/DOPO-HQ-conjugated flame-retardant composites was gradually enhanced, the glass fiber surface covered the carbon layer, and the pores on the surface of the carbon layer were reduced. Among them, the glass fibers serve as a skeleton support (Koppl et al., 2012). When the addition amount of DOPO-HQ was 14%, the LGF/PBT/DOPO-HQ-14-conjugated flame-retardant composites could form a dense carbon layer. This indicates that DOPO-HQ had a condensed phase flame-retardant effect on the system.

### Thermal stability analysis for LGF/PBT/DOPO-HQ-conjugated flame-retardant composites

**FIGURE 2:**
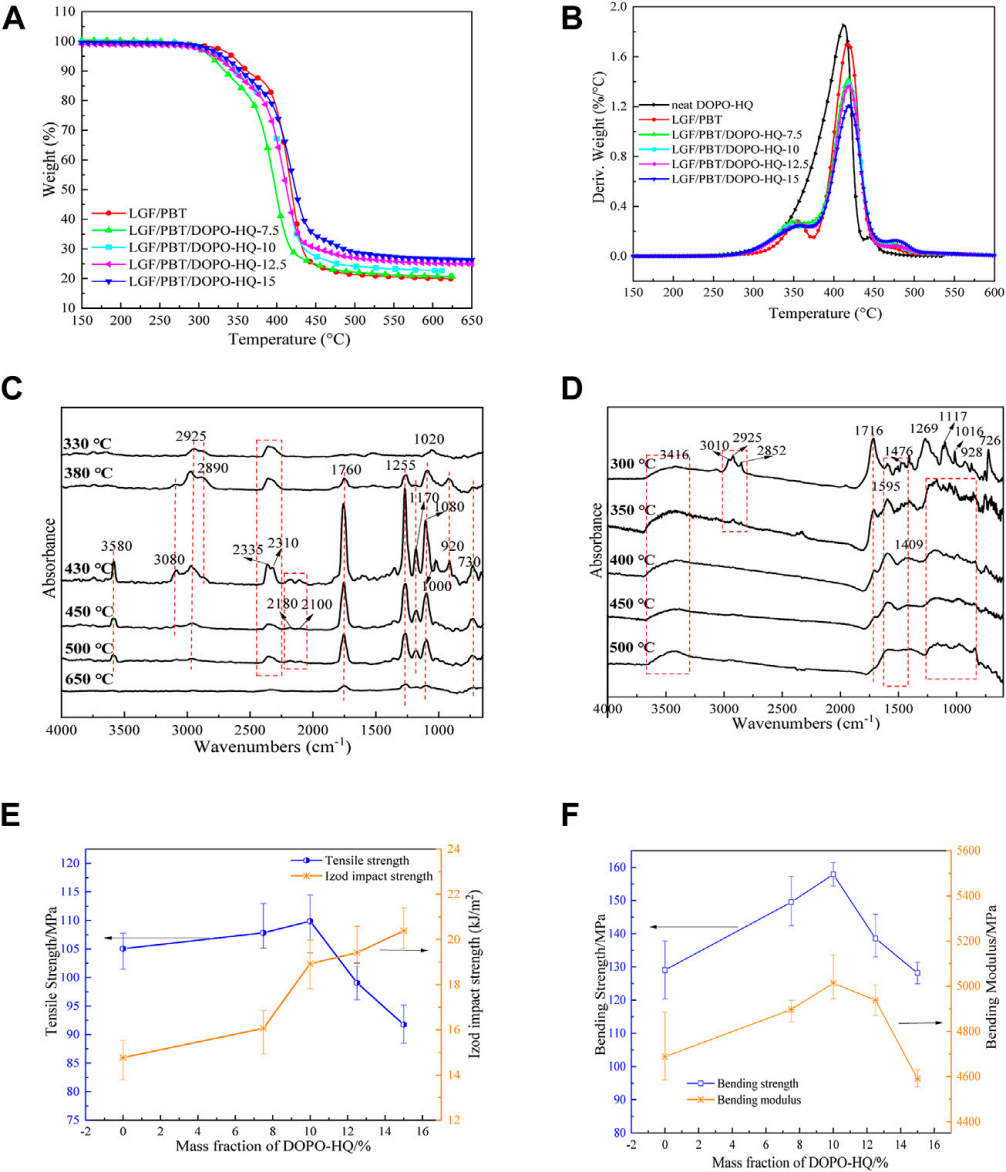
**(A,B)**: TG and DTG curves of LGF/PBT/DOPO-HQ-conjugated flame-retardant composites at the heating rate of 10°C/min in N_2_. **(C)** LGF/PBT/DOPO-HQ-conjugated flame-retardant composites and FTIR spectra of materials in the condensed phase, **(D)** the TG-FTIR results of LGF/PBT/DOPO-HQ-conjugated flame-retardant composites. **(E)** The tensile strength and Izod impact strength **(F)** the bending strength and bending modulus.

During the combustion process, the temperature of polymer materials generally exceeds 300°C. At this time, the thermal decomposition rate of polymer materials is greater than the diffusion rate of oxygen. Therefore, the combustion behavior of polymer materials is, in general, consistent with their thermal decomposition behavior (Zhan et al., 2015). In this article, the effect of DOPO-HQ on the thermal stability of LGF/PBT/DOPO-HQ-conjugated flame-retardant composites was investigated by TG, and the results are shown in Figures 2A,B. According to Figure 2A, the addition of DOPO-HQ to LGF/PBT composites shifted the initial decomposition temperature (T_5%_) of the conjugated flame-retardant composites to low temperatures and increased the residual amount. However, the T_5%_ of the conjugated flame-retardant composites gradually increased with the increase of DOPO-HQ addition. This may happen because DOPO-HQ is cross-linked with the matrix at this temperature, and the degree of cross-linking increased with the increase of DOPO-HQ content, which resulted in a gradual increase of T_5%_ of the conjugated flame-retardant composites. Meanwhile, according to Figure 2B, the main decomposition temperatures of DOPO-HQ and the matrix were the same, with Td values ranging from 300 to 450°C, and the maximum decomposition of the conjugated flame-retardant composites increased with the addition of DOPO-HQ. The maximum decomposition temperature peak of the conjugated flame-retardant composites gradually decreased and the maximum decomposition temperature was the same as that of LGF/PBT composites (i.e., about 429°C). With the increase of DOPO-HQ addition, the residual amounts of LGF/PBT/DOPO-HQ-7.5, LGF/PBT/DOPO-HQ-10, LGF/PBT/DOPO-HQ-12.5, and LGF/PBT/DOPO-HQ-15 increased to 7.62 wt%, 24.8 wt%, 39.5 wt%, and 43.3 wt%, respectively, compared to LGF/PBT. This may be due to the lower initial decomposition temperature of DOPO-HQ (T_5%_ 327.3°C), which decomposed earlier than the conjugated flame-retardant composites. This induced a lower initial decomposition temperature of the conjugated flame-retardant composites, which changed the decomposition pathway of the matrix and reduced the mass. The rate of loss promoted the formation of a carbon layer. These results indicate that DOPO-HQ helps to improve the flame-retardant properties of the system.

### Dynamic thermal decomposition analysis for LGF/PBT/DOPO-HQ-conjugated flame-retardant composites

According to Figure 2C, the LGF/PBT/DOPO-HQ-conjugated flame-retardant composites are in the initial thermal decomposition region, where the temperature is about 330°C. The main absorption peaks in the FTIR spectrum have aliphatic chains of methylene. (-CH_2_-) stretching vibration peaks, where the asymmetric stretching vibration peak of CH in the aliphatic chain is at 2925 cm^−1^, the symmetric stretching symmetry peak of CH in the aliphatic chain is at 2890 cm^−1^, and 1020 cm ^−1^ may be CC stretching vibration and covering PO stretching vibration. The absorption peak of CO_2_ (2335 cm^−1^) is also detected. When the temperature was further increased to about 380°C, the FTIR absorption peaks of the thermal decomposition products of the LGF/PBT/DOPO-HQ-conjugated flame-retardant composites were enhanced in intensity, and new absorption peaks were also generated, such as the benzene ring at 3080 cm^−1^. The stretching vibration peak of CH was the carbonyl stretching vibration peak with wave number 1760 cm^−1^, which may be the wave number 1250 cm^−1^ of PC and CO stretching vibration peaks, it is a PO stretching with wave number 1080 cm^−1^ vibrational peak. The bending vibrational peak of CH of the benzene ring has a wave number of 730 cm^−1^. In addition, it may be the aromatic organophosphorus composites with wave numbers of 920 cm^−1^, 1080 cm^−1^, and 1170 cm^−1^. These results are consistent with the relevant literature (Papkov et al., 2013). In other words, the thermal decomposition of the conjugated flame-retardant composites may release aromatic esters and aromatic organophosphorus complexes. This suggests that DOPO-HQ is added to the matrix, which contributes to the early decomposition of phosphorus groups in the conjugated flame-retardant composites. Therefore, it is advantageous for gas-phase flame retardants. In the maximum thermal decomposition region of the LGF/PBT/DOPO-HQ-conjugated flame-retardant composites (i.e., about 450°C), the initial decomposition products of the conjugated flame-retardant composites had the strongest peak intensity, and the absorption peaks of -OH stretching vibration (3580 cm^−1^) and CO (2180 cm^−1^ and 2100 cm^−1^) were also detected. However, the peak intensities of FTIR decreased significantly with the further increase of the thermal decomposition temperature. When the temperature reaches 650°C, the peaks at 3580 cm^−1^ (-OH), 2900 cm^−1^ (CH), and 1170 cm^−1^ (P=O stretching vibration), as well as CO2 (2335 cm^−1^) and CO (2180 cm^−1^ and 2100 cm^−1^) disappear. These behaviors suggest that the thermal decomposition products of DOPO-HQ may have PO_2_- and PO-, which can trap H- and OH, respectively, and effectively suppress the flame and form P-OH. Therefore, DOPO-HQ is in the homemade PBT matrix. Furthermore, it acts as a flame-retardant gas phase.

Figure 2D shows the FTIR spectra of the condensed phase of the LGF/PBT/DOPO-HQ-conjugated flame-retardant composites. According to Figure 2D, there are different absorption peaks in the initial thermal decomposition region of the LGF/PBT/DOPO-HQ-conjugated flame-retardant composites. These absorption peaks are about 3416 cm^−1^ (-OH), 2995, 2925 and 2852 cm^−1^ (CH), 1716 cm^−1^ (C=O), 1400 to 1600 cm^−1^ (benzene ring), 1269 cm^−1^ (CO), and 1117 to 1060 cm^−1^ (P=O and PO). However, as the thermal decomposition temperature increases, the intensity of the absorption peaks at 1720 cm^−1^ and 1260 cm^−1^, and then decreases until they disappear, which is CO2 (2335 cm^−1^) at 650°C in Figure 2C. Corresponding to the disappearance of the peaks of CO (2180 cm^−1^ and 2100 cm^−1^), the absorption peaks from 1400 cm^−1^ to 1600 cm^−1^ and 1000 cm^−1^ to 1200 cm^−1^ also gradually broadened. However, there is still a broad absorption peak near 3416 cm^−1^, which may be the associated absorption peak of hydroxyl group. Combined with the aforementioned findings, these behaviors suggest that the LGF/PBT/DOPO-HQ-conjugated flame-retardant composites have a polyaromatic ring structure and a polyphosphate structure in the condensed phase after combustion. This is consistent with the relevant reports in the literature (Koppl et al., 2012; Qian et al., 2015) and indicates that DOPO-HQ has a certain condensed phase flame-retardant effect on the PBT matrix.

### Mechanical properties of LGF/PBT/DOPO-HQ-conjugated flame-retardant composites

The mechanical properties of modified composites are an important goal for practical production applications. However, the addition of flame retardants to the matrix usually leads to a decrease in the mechanical properties of the composites. Figures 2E,F show the mechanical properties of LGF/PBT/DOPO-HQ-conjugated flame-retardant composites. From Figures 2E,F, it can be seen that the tensile strength, flexural strength, and flexural modulus of the conjugated flame-retardant composites first increased and then decreased with the increase of DOPO-HQ addition, while the impact strength gradually increased. When 15% DOPO-HQ was added, the tensile strength, flexural strength, and flexural modulus of LGF/PBT-15 system decreased by 12.64, 0.7, and 2.12%, respectively, while the impact strength increased by 27.53%. This may be due to the chemical reaction between DOPO-HQ as a reactive conjugated flame retardant and the matrix. Therefore, the mechanical properties of the conjugated flame-retardant composites were still good when V-0 was reached.

## Conclusion

In this article, the conjugate flame-retardant DOPO-HQ was applied to PBT. The performance test of the conjugate flame retardant shows that the vertical burning time, PHRR, HRR, THR, MAHRE, and FIGRA of LGF/PBT/DOPO-HQ conjugate flame-retardant composites decreased with the increase of DOPO-HQ content, and the LOI showed a gradual increasing trend. When 14% of DOPO-HQ was added, the flame-retardant performance of the conjugated flame-retardant composites reached UL-94 V-0, and the PHRR, HRR, and THR of the flame-retardant system decreased by 56.5, 45.9, and 32.6%, respectively, while the LOI increased to 26.4%. This indicates that DOPO-HQ has a good flame-retardant effect on LGF/PBT composites.

LGF/PBT/DOPO-HQ-conjugated flame-retardant composites release PO_2_- and PO-, which can capture H- and OH, and form aromatic esters and aromatic phosphorus compounds (including P-OH structure). Consequently, the free-radical segment of the conjugated flame-retardant composites is terminated during combustion, the flame is effectively suppressed, and the flame-retardant performance is enhanced. In addition, DOPO-HQ migrates, and the LGF/PBT/DOPO-HQ-conjugated flame-retardant composites increase the phosphorus content in the carbon layer after combustion. This indicates that DOPO-HQ has a cohesive phase flame-retardant effect on LGF/PBT composites. As the DOPO-HQ addition is increased, the denseness of the carbon layer formed after combustion of LGF/PBT/DOPO-HQ-conjugated flame-retardant composites is increased. FTIR analysis of the carbon layer showed that the carbon layer of LGF/PBT/DOPO-HQ-conjugated flame-retardant composites had multi-aromatic rings and multi-phosphate after combustion. This indicates that DOPO-HQ exerted a condensed phase flame-retardant effect on LGF/PBT composites. In conclusion, DOPO-HQ is a promising flame-retardant additive for PBT, mainly as a gas-phase flame retardant, especially when it is used together with LGF.

## Data Availability

The raw data supporting the conclusion of this article will be made available by the authors, without undue reservation.
